# Dataset for the cost estimation of the building envelope in the Northern Region of Morocco

**DOI:** 10.1016/j.dib.2024.110048

**Published:** 2024-01-11

**Authors:** Ettoumi Youssef, Romani Zaid, El Hadad Imad, Meftah Khalid

**Affiliations:** a Laboratory of the Built Environment and Landscape (LaBEL), National School of Architecture of Tetouan, Morocco; b Laboratory of Research in Economics and Management of Organisations (LEMO), UIT- Kénitra, Morocco; c Laboratory of Research in Management (LRM), University Med V, Rabat, Morocco

**Keywords:** Construction economics, Insulation materials, Material prices, Ratios, Budget Overrun, Phases of construction

## Abstract

Today, in the great majority of buildings in Morocco, the exterior envelope is not insulated and is therefore not performant from a thermal point of view. This data article describes and analyses a dataset of ratios (cost by square meter that we elaborated for the estimation of the cost of buildings at an early phase of construction. The ratios also allow to take into consideration the generated cost caused by the integration of insulation materials.

Based on the primary data that were collected by the economic observatory of the provincial delegation of the Ministry of Urbanism, Habitat, Territory and City Policy, we performed our analysis to come up with our own secondary data which are the ratios of the cost (DH/m^2^). We focused on four elements of the building envelope namely: the external walls, the windows, the roof, and the floor. The ratios are of a great significance for construction economists and architects allowing them to make cost trade-off between different technical solutions throughout the stages of the building thus, potentially leading to a life cycle cost optimization

Specifications Table

**Table:** 

Subject	Economics
Specific subject area	Construction Economics
Type of data	Tables Figures Graphs
How the data were acquired	Data was retrieved from a survey conducted by the Economic Observatory of the provincial delegation of the Ministry of Urbanism and Territorial management in the city of Tetouan.
Data format	Mixed: Raw, Processed and Analysed.
Description of data collection	Data is secondary; it is the result of primary data that was gathered via a survey conducted by the provincial delegation of the Ministry of Urbanism, Territorial management and City Policy in the city of Tetouan. It is an annual survey that started in 2005 and it has been updated every year since. The firms are operating in the northern region of Morocco and specialize in selling construction materials. The fieldwork was mainly carried out through direct interviews with the sources.
Data source location	City:Tetouan, Country: Morocco. Latitude: 35.588899 Longtitude: −5.3625516
Data accessibility	Raw Data and analysed Data have been deposited in a free public depository: Mendeley Data. They are accessible via the following two links below: The direct URL link to Raw Data: https://data.mendeley.com/datasets/gtrc2r2gzd/1 DOI: 10.17632/gtrc2r2gzd.1 The direct URL link to Analysed Data: https://data.mendeley.com/datasets/894wyrck2j/1 DOI: 10.17632/894wyrck2j.1

## 1 Value of the Data

• The dataset could be used by construction economists and architects to help estimate the cost of buildings at an early stage of the construction process (the design phase).
• The dataset gives details on the additional cost generated by the integration of the thermal insulation of the building envelope.
• The dataset could be used by researchers to elaborate synthetic indexes to help analyse and forecast the market trajectories and price trends in the future.
• The dataset presents the long term and recent evolution of the prices of building materials in the northern region of Morocco.

## 2 Objective

In many parts of the world and mostly in developing countries such as Morocco, the cost estimation of construction projects is still imprecise and vague. This causes cost estimation problems and a lack of vision for all the project stakeholders. Indeed, architects, projects owners and general contractors do not have a predefined and precise ratio for specific elements of the building, moreover there are no specific ratios that take into account the additional cost generated by the integration of insulation materials. This makes the determination of the initial construction budget very difficult.

The objective of this article is twofold: first, it aims to facilitate the estimation of the cost of the building envelope at an early phase of the construction process by developing ratios (cost by square meter) for 4 different parts of the building. Second, the article aims at estimating the additional cost generated by the integration of insulation materials for the same 4 parts of the building envelope namely: External walls, Roof, Floor and Windows.

## 3 Data Description

This data article describes a dataset of ratios for the cost of different parts of the building envelope to be used in the estimation of the building's total initial cost [1,2]. Indeed, the external walls of the building envelope are in the majority of cases built of bricks with cement plasters, the roof is composed of a screed, a hollow body floor and an interior cement coating. The floor consists of a tile, a screed and a reinforced concrete slab. Finally, the windows are single glazed with aluminium frames [3].

The idea is to develop ratios cost by square metre (DH/m^2^) that take into account the integration of thermal insulation and high building energy performance [4,5] : so that from the design phase, one could be able to estimate the cost of a building with a high-performant envelope, that is well insulated. The precalculated ratios are crucial as they allow gaining time and avoiding budget overruns.

**Image 1:**
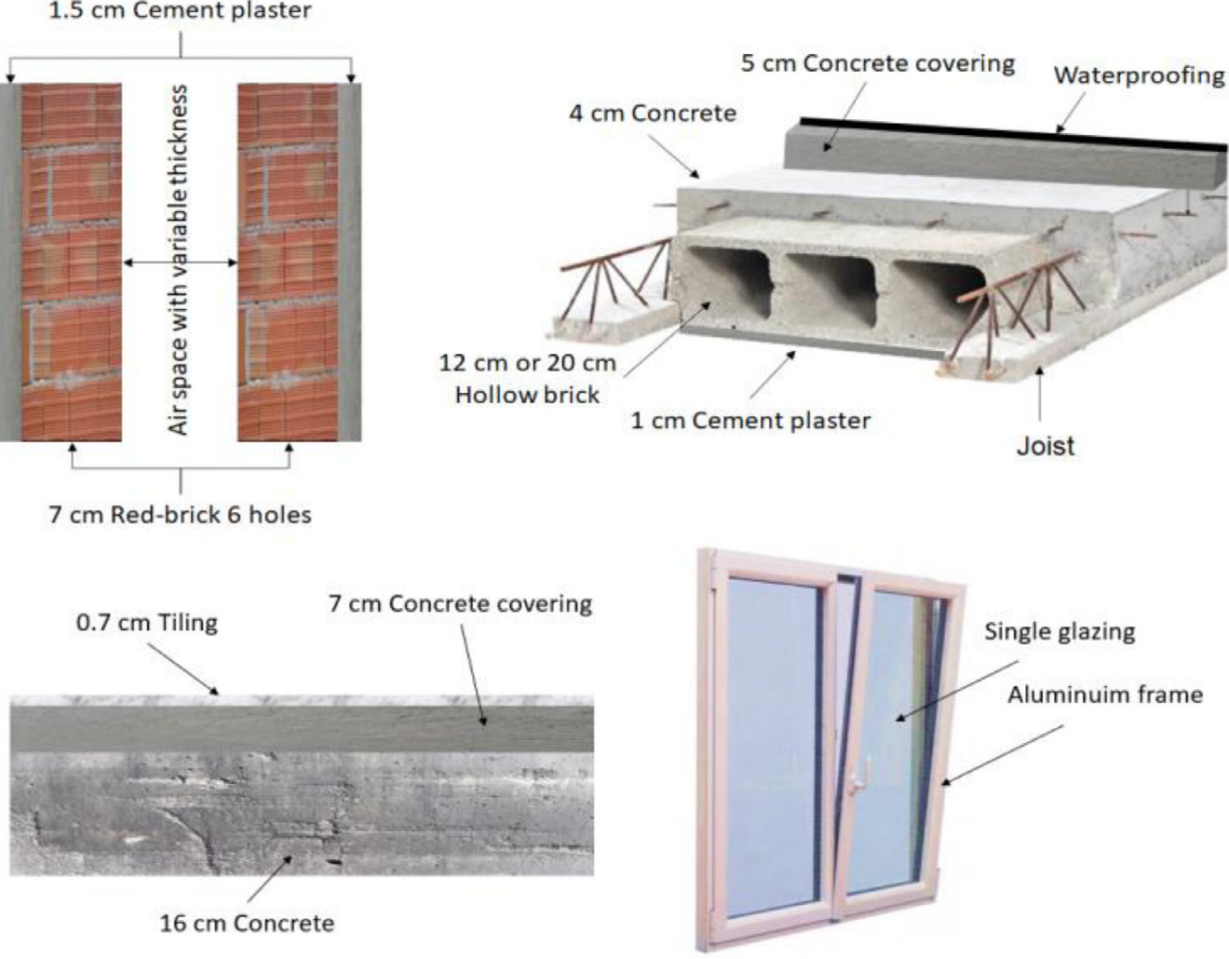
The four parts of the building envelope studied.

**Fig 1:**
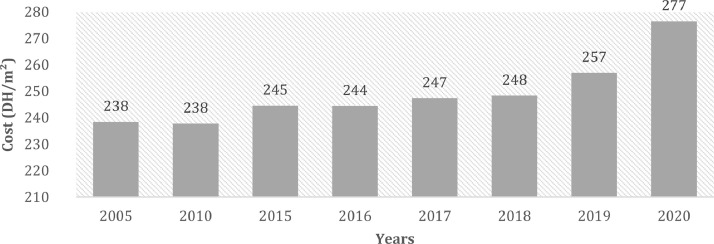
The evolution of the cost of external wall (DH/m^2^) without insulation materials between 2005 and 2020.

**Fig 2:**
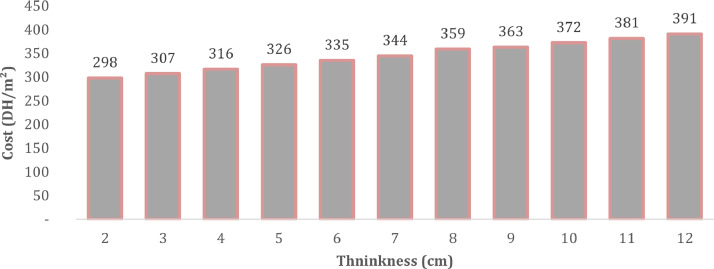
The cost of external wall (DH/m^2^) after the integration of insulation materials of different thicknesses ranging from 2 centimetres (cm) to 12 cm in the year 2020.

Image 1 describes the 4 elements object of our study. Fig. 1 describes the evolution of the cost of external walls (DH^1^/m^2^) without the insulation materials. Fig. 2 describes the cost of external walls (DH/m^2^) after the addition of insulation materials.

**Fig 3:**
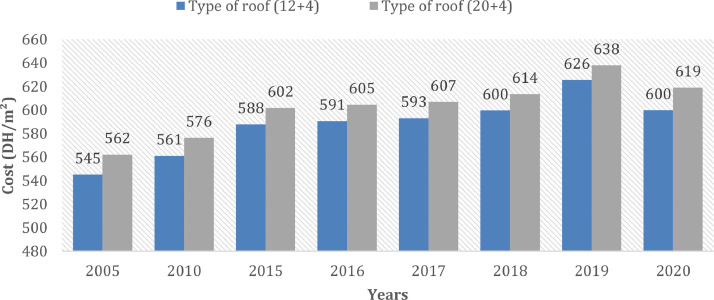
The evolution of the cost (DH/m^2^) of the roof without insulation between 2005 and 2020.

**Fig. 4:**
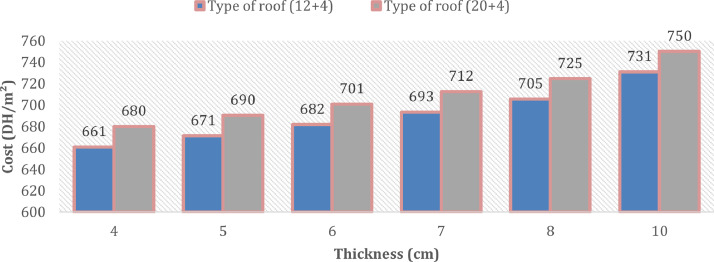
The evolution of the cost (DH/m^2^) of the external roof with multiple insulation thicknesses ranging from 4 cm to 10 cm in the year 2020.

**Fig 5:**
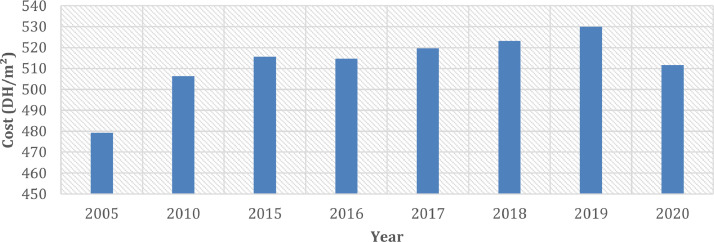
The evolution of the cost (DH/m^2^) of the floor without insulation between 2005 and 2020.

**Fig 6:**
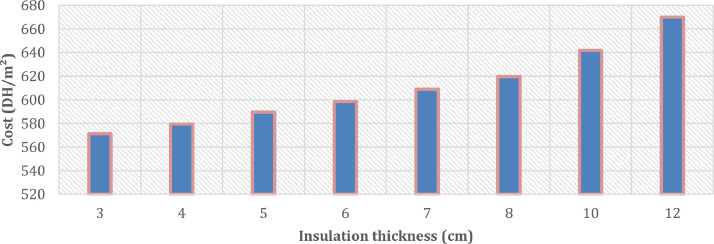
The evolution of the cost of the floor (DH/m^2^) with multiple insulation thicknesses ranging from 3 cm to 12 cm in the year 2020.

Fig. 3 presents the evolution of the cost (DH/m^2^) of the roof without insulation in the case for two types of roof slab. Fig. 4 describes the cost of the external roof in the case of two types of insulated roof slab. Fig. 5 describes the evolution of the cost (DH/m^2^) of the floor without insulation between 2005 and 2020. Fig. 6 presents the evolution of the cost of the floor (DH/m^2^) with multiple insulation thicknesses ranging from 3 cm to 12 cm in the year 2020.

**Fig 7:**
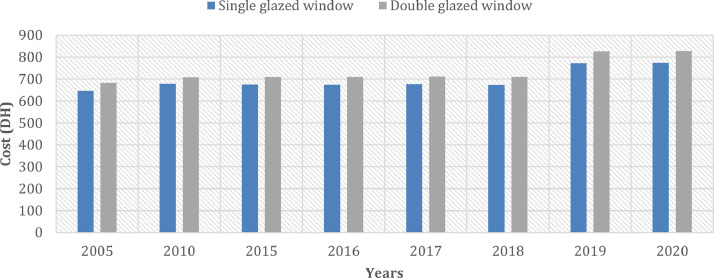
The evolution of the cost of windows (DH/m^2^) in the case of single- and double-glazed windows.

Fig. 7 describes the evolution of the cost of windows (DH/m^2^) in the case of single and in the case of more performing windows (double glazing windows).

Noting that for the four elements studied, the cost for different thicknesses of the insulation was estimated only for the year 2020.

Finally, Table 1 represents a comparison of the cost (DH/m^2^) of the four different parts of the building envelope in the northern region of Morocco in 2020 before and after the integration of insulation materials.

**Table 1:** Prices of the 4 building elements before and after the integration of insulation materials^2^ in 2020.

Element of the building	Prices(DH/m^2^) in 2020	
	Without insulation materials	With the integration of insulation materials
External walls	277	391
Windows	774	827
Roof (12+4) Roof (20+4)	600 618	730 750
Floor	512	670

2 The table above presents a summary of the costs for the four elements of the building envelope with and without insulation materials. Only the prices of the highest thickness of the insulation were considered.

The image above technically describes the 4 main parts of the building's envelope, namely the external walls, the floor, the roof and the windows. For each one, we integrated additional elements (insulation materials): For the walls, it is the addition of an expanded polystyrene in the air gap, for the floor, it is the extruded polystyrene insulation between the screed and the concrete slab, for the roof it is the external insulation using the extruded polystyrene and finally with regard to the windows it is a double-glazed windows instead of a single glazed-window.

The figure above shows the evolution of the cost of an exterior wall in the northern region of Morocco. This cost increased by 14% between 2005 and 2020 to reach 277 DH/m^2^.

In Morocco the new thermal building regulation requires the use of insulation materials in the walls [6]. We present in Fig. 2 below, the cost of the wall with the integration of the expanded polystyrene in the air gap as an insulation material. The figure describes the price of the wall with different insulation thicknesses (from 2 cm to 12 cm) for the year 2020.

The evolution of the cost according to the thickness of the insulation is almost linear. The additional cost in the case of 2 cm thick insulation is 22 DH / m^2^, which represents an increase of 7.6%. The cost difference between 2 cm thickness and 12 cm thick insulation is 93 DH/m^2^.

Regarding the roof, two types of hollow body roof were studied as shown in the figure above. The difference between the two lies in the height of the slabs which may be different depending on the structural calculation. It was noted that the price of hollow body slabs changed between 2005 and 2019 with a rise of 15% and 13% for the case of the “12 + 4” and “20 + 4” ROOF respectively. On the other hand, a decrease was observed between 2019 and 2020 which is of the order of 26 DH/m^2^ and 19 DH/m^2^ respectively.

As part of thermal regulations, insulation can be provided for the roof. Several thicknesses of extruded polystyrene insulation have been applied for simulation. For the two types of the cases proposed, the price increases by 61 DH/m^2^. The additional cost between 4 cm and 10 cm of roof insulation is around 70 DH/m^2^.

Regarding the floor, we see a non-linear evolution of the price and an increase of 7% in 15 years. In addition, a price drop was noticed between 2019 and 2020 of 3.5%.

In the case of the addition of 3 cm thermal insulation using the extruded polystyrene in the floor, the price goes from 479 DH/m^2^ to 571 DH/m^2^ and therefore an increase of 19%. Knowing that thermal insulation of the floor is not mandatory in certain climates and thermal conditioning of buildings, over-insulation of the lower floor is not recommended. It should be noted that the change from 3 cm of insulation to 12 cm generates an additional cost of 17%.

Concerning the case of the windows, in the Moroccan construction market, two types of windows are mainly used: single glazing and double glazing with aluminium frames. Between 2005 and 2018 the price of windows was almost constant. From 2019, the price increased by 98.8 DH/m^2^ which represents an evolution of 15%. It should be noted that the difference between the price of single glazing and double glazing is 5 DH per meter square in 2020.

## 4 Experimental Design, Materials and Methods

### 4.1 The sample

The choice of the companies subject to the survey was made using the stratified selection method according to the three following criteria: the type, the size and the legal status of the company.

These firms were spatially distributed in 2020 as follows: 47% for the city of Tangier, 24% for that of Tetouan, 22% for Al Hoceima city, 6% for Larache city and only 1% for the city of Chefchaouen.

The primary data used consists of the price of a set of building materials in the main cities of the Northern region of Morocco: More precisely It concerns: 33 items for the structural work and 87 items for the finishing work (120 items in total). This set of materials is structured in the form of nested sub-assemblies that are hierarchized based on the chronological succession in the construction process.

### 4.2 The method

The fieldwork was mainly carried out through direct interviews with the sources. For some responders whose responsiveness was reduced, alternative modes have been applied, including: Telephone calls for confirmation of certain missing information, in coordination with the back office set up to support the field team.

Based on the collected data, we performed our own analysis to come up with ratios (cost by square meter) that take into account the integration of building insulation materials to the 4 different parts of the building's envelope. For each part, we integrated different thicknesses: from 2 cm to 12 cm. In order to have a precise estimation, we also took into account the cost of labour necessary for the construction of each building part.

## Limitations

Not applicable.

## Ethics Statement

The current work does not involve human subjects, animal experiments, or any data collected from social media platforms.

## CRediT authorship contribution statement

**Ettoumi Youssef:** Conceptualization, Methodology, Data curation, Visualization, Writing – original draft, Writing – review & editing. **Romani Zaid:** Conceptualization, Methodology, Data curation, Visualization, Writing – original draft, Writing – review & editing. **El Hadad Imad:** Data curation, Writing – original draft. **Meftah Khalid:** Data curation, Writing – original draft.

## Data Availability

Raw Data of the prices of construction materials in the Northern region of Morocco (2005-2020) (Original data) (Mendeley Data).

## Footnotes

1 *The Dirham (DH) is the Moroccan currency. 1 US dollar is the equivalent of 10.13DH as of the 8^th^ December 2023.*

## References

[1] Abdou N., Mghouchi Y.E.L, Hamdaoui S., Asri N.E.L, Mouqallid M. (2021). Multi-objective optimization of passive energy efficiency measures for net-zero energy building in Morocco. Build. Environ..

[2] Lu K., Jiang X., Yu J., Tam V.W.Y., Skitmore M. (2021). Integration of life cycle assessment and life cycle cost using building information modeling: a critical review. J. Clean. Prod..

[3] Romani Z., Draoui A., Allard F. (2015). Metamodeling the heating and cooling energy needs and simultaneous building envelope optimization for low energy building design in Morocco. Energy Build..

[4] Raji B., Tenpierik M.J., Van Den Dobbelsteen A. (2015). The impact of greening systems on building energy performance: a literature review. Renew. Sustain. Energy Rev..

[5] Laghmich N., Romani Z., Lapisa R., Draoui A. (2021). Numerical analysis of horizontal temperature distribution in large buildings by thermo-aeraulic zonal approach. Build. Simul..

[6] AMEE, Le Règlement Thermique de Construction au Maroc, Morocco, 2014. https://www.amee.ma/sites/default/files/inlinefiles/Reglement_thermique_de_construction_au_Maroc.pdf.

